# Mitochondrial ATP Synthase and Mild Uncoupling by Butyl Ester of Rhodamine 19, C4R1

**DOI:** 10.3390/antiox12030646

**Published:** 2023-03-04

**Authors:** Ljubava D. Zorova, Irina B. Pevzner, Ljudmila S. Khailova, Galina A. Korshunova, Marina A. Kovaleva, Leonid I. Kovalev, Marina V. Serebryakova, Denis N. Silachev, Roman V. Sudakov, Savva D. Zorov, Tatyana I. Rokitskaya, Vasily A. Popkov, Egor Y. Plotnikov, Yuri N. Antonenko, Dmitry B. Zorov

**Affiliations:** 1 A.N. Belozersky Institute of Physico-Chemical Biology, Lomonosov Moscow State University, 119992 Moscow, Russia; 2 V.I. Kulakov National Medical Research Center of Obstetrics, Gynecology and Perinatology, 117997 Moscow, Russia; 3 Bach Institute of Biochemistry, Research Center of Biotechnology of the Russian Academy of Sciences, 119071 Moscow, Russia; 4 N.N. Blokhin Russian Cancer Research Center, 115478 Moscow, Russia; 5 Faculty of Bioengineering and Bioinformatics, Lomonosov Moscow State University, 119992 Moscow, Russia

**Keywords:** mitochondria, mitochondria-targeted drugs, antioxidants, mild uncoupling, ATP synthase, Complex V

## Abstract

The homeostasis of the transmembrane potential of hydrogen ions in mitochondria is a prerequisite for the normal mitochondrial functioning. However, in different pathological conditions it is advisable to slightly reduce the membrane potential, while maintaining it at levels sufficient to produce ATP that will ensure the normal functioning of the cell. A number of chemical agents have been found to provide mild uncoupling; however, natural proteins residing in mitochondrial membrane can carry this mission, such as proteins from the UCP family, an adenine nucleotide translocator and a dicarboxylate carrier. In this study, we demonstrated that the butyl ester of rhodamine 19, C4R1, binds to the components of the mitochondrial ATP synthase complex due to electrostatic interaction and has a good uncoupling effect. The more hydrophobic derivative C12R1 binds poorly to mitochondria with less uncoupling activity. Mass spectrometry confirmed that C4R1 binds to the β-subunit of mitochondrial ATP synthase and based on molecular docking, a C4R1 binding model was constructed suggesting the binding site on the interface between the α- and β-subunits, close to the anionic amino acid residues of the β-subunit. The association of the uncoupling effect with binding suggests that the ATP synthase complex can provide induced uncoupling.

## 1. Introduction

The coupling of oxidation and phosphorylation is a tight cooperation of the activity of proton pumps with ATP synthase machinery [1,2,3,4]. In addition, in some prokaryotic organisms such coupling exists between sodium pumps and ATP synthase utilizing sodium gradients [5,6,7]. On the other hand, the term “uncoupling” of oxidative phosphorylation meaning separation of oxidation from phosphorylation is also under wide use being organized by two distinct mechanisms. One of those is hidden in the coupling mechanism and reflects the loss of tightness/efficiency of this mechanism, and another one also compromising the proton potential stands aside of the proton pumps and ATP synthase providing an ion leak through either phospholipids or proteins residing in the coupling membrane. Whatever the mechanism, ultimately both uncoupling mechanisms yield the same result—lower ATP production per protons pumped through the coupling membrane [4].

There are two complementary theories of uncoupling. One process is a passive proton transfer by chemical uncouplers discharging the proton gradient. Within such mechanism, the uncoupler must have a chemical group binding a proton and being highly lipid-soluble, such complex is transferred through the membrane with subsequent dissociation of the complex, ultimately providing a proton transfer from a more acidic compartment to a less acidic one. The proton current through the artificial membranes caused by chemical uncouplers was demonstrated in classic studies many years ago [8,9].

The second uncoupling mechanism was proposed based on data demonstrating the involvement of proteins residing in the inner mitochondrial membrane. The most studied proteins belong to the class of “uncoupling proteins” (UCPs) and, historically, the earliest uncoupling protein was found in the mitochondria of brown fat (UCP1, [10]). In addition to UCP1, a similar set of its orthologs were found in different tissues with a tentative uncoupling function [11].

However, it turned out that the range of uncoupling proteins is not limited only to UCP family. Later, it has been found that proton transport across mitochondrial membranes can be significantly slowed down by chemicals blocking the activity of other protein components of the inner mitochondrial membrane. Within this set of data, we can recall the results with atractylate/carboxyatractylate which are specific inhibitors of adenine nucleotide translocator (ANT) and diminished the uncoupling efficiency of fatty acids [12]. Later, the loss of the uncoupling activity of fatty acids has been demonstrated after inhibiting the activity of mitochondrial dicarboxylate carrier [13]. Both results have been interpreted as a support of a protein-mediated mechanism of uncoupling. Recently, electrophysiological experiments confirmed the direct involvement of ANT in the fatty acid and uncoupler-mediated uncoupling [14,15,16]. We must admit that such dual mechanism including passive and mediated transfer is not unique and, for example, it is valid for a water transfer through the membrane: although lipid membranes are water permeable, aquaporins harboring the phospholipid membranes facilitate the transmembrane water transport [17].

Less interpretable was the result that 6-ketocholestanol abolished the uncoupling activity of the strongest uncouplers [18]. However, besides the direct 6-ketocholestanol effect on the lipid bilayer membrane this also suggested the presence in the inner mitochondrial membrane of proteinaceous components providing mediated proton transfer [19]. As we have already mentioned, the best-known inner membrane proteins having a common name, UCPs might potentially carry this mission. Indeed, UCP1 orthologs (UCP1-5) in mammalian mitochondria from different tissues initially were proposed to also provide an uncoupling, i.e., thermogenic role which was obvious in brown fat, but considering their low abundance this idea was highly argued [20,21,22,23]. Still, one of the most essential functions of UCPs has been proposed to prevent the production of high levels of membrane potential. The latter was suggested to be associated with high level of reactive oxygen species (ROS) production due to exponential dependence of ROS production on the values of the mitochondrial membrane potential [24]. Since in mitochondria having high mitochondrial membrane potential, production of both ATP and ROS is high, it has been found to be reasonable to regulate the production of ROS by lowering the membrane potential still keeping its values above the phosphorylation potential [25]. Fatty acids due to their limited ability to uncouple oxidative phosphorylation were named as intrinsic uncouplers providing moderate (mild) uncoupling, thus keeping the production of ROS low but optional for intracellular signaling level. The reasonable balance between the level of ATP and ROS production has been suggested to be a requisite for a healthy life [26].

There is a set of chemicals demonstrating mild uncoupling ability. Considering a general proof that mild uncoupling is therapeutically beneficial [27,28,29,30,31,32,33] although the protective mechanisms stay mainly unresolved, this study aimed to resolve this mechanism basing on assumption that mild uncouplers bind to specific mitochondrial proteins ultimately providing moderate ion leak through the mitochondrial membrane.

In early studies, it has been shown that a number of mitochondria targeted agents from the SkQ family could potentially be used as therapeutic agents associated with mild mitochondrial uncoupling [29]. Over the past 15 years, a wide range of physico-chemical and biological properties of these derivatives has been comprehensively explored [25,26,27,28,29,30,31,32,33,34,35,36,37,38,39,40].

**Figure 1:**
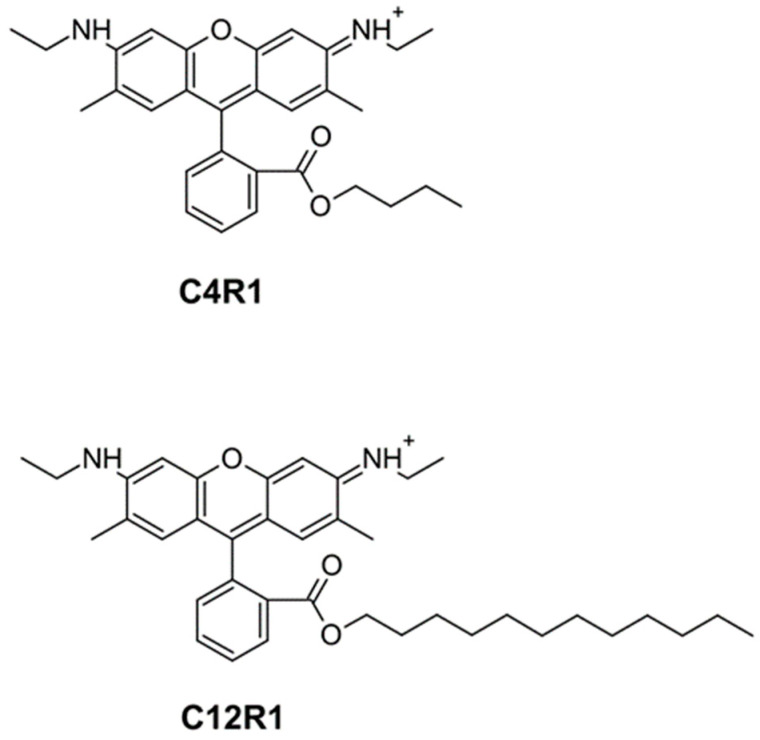
Chemical structure of C4R1 (butyl ester of rhodamine 19) and C12R1 (dodecyl ester of rhodamine 19).

In this study, the final goal was to elucidate the mechanism of the uncoupling potency of the butyl ester of rhodamine 19 (C4R1) which, as previously shown [28], has a greater effect than the more hydrophobic analog dodecylrhodamine 19 (C12R1, see structures in Figure 1), which indirectly indicated different mechanisms of uncoupling by agents similar in structure. Of note, the ethyl ether of rhodamine 19 (rhodamine 6G, C2R1) was long known to exhibit low uncoupling activity [41,42]. It has been shown previously that C12R1 has higher protonophoric activity compared to C4R1 on artificial lipid membranes [39,43].

## 2. Materials and Methods

### 2.1. Synthesis of Rhodamine Derivatives

Derivatives of rhodamine 19 were synthesized in A.N.Belozersky Institute by Natalia V. Sumbatyan and Galina A. Korshunova, as described in [38,40].

### 2.2. Isolation of Rat Liver Mitochondria

Rat liver and heart mitochondria were isolated using the standard method of differential centrifugation [44] in a medium containing 250 mM sucrose, 5 mM MOPS, 1 mM EGTA, and bovine serum albumin (0.5 mg/mL), pH 7.4. The final washing was performed in a medium of the same composition but without albumin. Protein concentration was determined using the biuret method. Handling of animals and experimental procedures with them were conducted in accordance with the international guidelines for animal care and use and were approved by the Institutional Ethics Committee of Belozersky Institute of Physico-Chemical Biology at Moscow State University.

### 2.3. Mitochondrial Respiration

Respiration of isolated rat liver mitochondria was measured using a standard polarographic technique with a Clark-type oxygen electrode (Strathkelvin Instruments, Motherwell, UK) at 25 °C using the 782 system software. The incubation medium contained 250 mM sucrose, 5 mM MOPS, 1 mM MgCl_2_, 1 mM KH_2_PO_4_ and 0.2 mM EGTA, pH 7.4. The mitochondrial protein concentration was 0.8 mg/mL. Oxygen uptake was expressed as nmol/min mg protein.

### 2.4. Isolation of ATP Synthase Complex (FoF1, Complex V)

FoF1 was purified from isolated rat heart mitochondria according to the manufacturer protocol using ATP Synthase Immunocapture Kit (Abcam, Waltham, MA, USA, ab109715).

### 2.5. Electric Potential of Membranes of Bacillus subtilis with DiS-C3-(5)

The *Bacillus subtilis* strain BR151 was used in experiments. *Bacillus subtilis* cells were grown in Luria Bertani (LB) broth overnight at 37 °C in an incubator shaker at 210 rpm. The overnight culture reached A600 (absorbance) of 1.5. Cells were diluted 1:20 in the buffer containing 100 mM KCl, 10 mM Tris, pH 7.4. The fluorescence reading of DiS-C3-(5) was monitored by using a Panorama Fluorat 02 spectrofluorimeter (Lumex, Russia), with an excitation wavelength of 622 nm and an emission wavelength of 690 nm.

### 2.6. Binding C4R and C12R1 with Mitochondria and Bacillus subtilis Elucidated by 1D Electrophoresis

Mitochondrial samples, their fractions and preparations of purified complex V were mixed with a 4x sample buffer containing 0.125 M Tris-HCl (pH 6.8), 4% sodium dodecyl sulfate, 40% glycerol, 0.05% bromophenol blue, and 10% 2-mercaptoethanol, boiled for 5 min and applied to Tris-glycine polyacrylamide gel (40 μg per well), having previously determined the concentration of total protein using a set of bicynchoninic acid (Sigma, Burlington, MA, USA). Electrophoresis was carried out at 20 mA until the stain came out of the separating gel. At the end of electrophoresis, the fluorescence in the gel was visualized using the FUJIFILM FLA3000 PhosphorImager at an excitation wavelength of 532 nm.

To study the binding to the probe after electrophoretic separation of proteins, the gel was incubated with a solution of 20 µM C4R1 for 30 min at a constant rocking of a gel. After that, the gel was washed in a buffer containing 0.375 M Tris-HCl pH 8.8. Fluorescence was evaluated using PhosphorImager at an excitation wavelength of 532 nm.

### 2.7. Two-Dimensional Gel Electrophoresis

The protein extracts were fractionated by two-dimensional electrophoresis according to [45]. IEF was performed in glass tubes (2.4 × 180 mm) filled with 4% PAAG prepared in a 9 M urea solution containing 2% TritonX-100 and a 2% mixture of ampholines with pH 5–7 and pH 3.5–10 were used at a ratio of 4:1. The protein extracts (100–150 μL) were applied to the “acid border” of each gel column, and IEF was performed for 3 h at 1400 V/h (Model 175; Bio-Rad, Hercules, CA, USA). The fractionation in the second direction (SDS slab gel electrophoresis with the acrylamide concentration gradient 5–20%) was performed, as previously described [46].

### 2.8. Mass Spectrometry

Polyacrylamide gel stained with Coomassie Brilliant Blue was exposed to trypsin, as described earlier [46]. Gel pieces of about 2 mm^3^ were destained twice with 50 mM NH_4_HCO_3_, 40% aqueous acetonitrile solution, pH 7.5; dehydrated with 200 mL of 100% acetonitrile and rehydrated with 5 mL of the digestion solution containing 15 µg/mL sequencing grade trypsin (Promega) in 50 mM NH_4_HCO_3_, aqueous solution, pH 7.5. Digestion was carried out at 37 °C for 6 h. The resulting peptides were extracted with 5 mL of 0.5% TFA, 30% acetonitrile solution. A 1 μL aliquot of in-gel tryptic digest extract was mixed with 0.5 mL of 2,5-dihydroxybenzoic acid solution (30 mg/mL in 30% acetonitrile, 0.5% TFA) and left to dry on the stainless-steel target plate. MALDI-TOF MS analysis was performed on an UltrafleXetreme MALDI-TOF-TOF mass spectrometer (Bruker Daltonik, Bremen, Germany). The MH+ molecular ions were measured in a reflector mode; the accuracy of the monoisotopic mass peak measurement was within 50 ppm. Mass spectra were processed with the FlexAnalysis 3.2 software (Bruker Daltonik, Germany). Protein identification was carried out by peptide fingerprint search with the use of Mascot software version 2.3.02 (Matrix Science) through the Home SwissProt protein database. One missed cleavage, Met oxidation and Cys-propionamide were permitted. Protein scores greater than 70 were considered to be significant (*p* < 0.05).

### 2.9. Molecular Docking

The Swiss-Model service was used to model the structures of ATP synthases in humans, rats, *E. coli* and *B. subtilis* by homology. Further, it became necessary to scan the surface of the subunit by molecular docking methods in order to identify the intended binding site, considering necessity to exclude the interaction surfaces sterically closed by neighboring subunits. For this, the resulting structure of each subunit used in the work was embedded in the extra-membrane part of the yeast ATP synthase complex. Using the Gromacs software package, the point charges of the resulting complex were obtained, which are necessary for calculating electrostatic interactions.

The structure of the Rhodamine 6G which is very similar to the molecule of C4R1 and was available from the QacR(E120Q) structure (https://www.wwpdb.org/pdb?id=pdb_00003br6, assessed on 1 December 2022). The charges for the molecule were obtained using the tppmktop service (http://erg.biophys.msu.ru/tpp/, assessed on 1 December 2022) and the LigParGen service (http://zarbi.chem.yale.edu/ligpargen/, accessed on 1 December 2022).

The Autodock-Vina program was used to calculate the most optimal position of the ligand in the volume of a rectangle of certain dimensions (the dimensions of the intended binding site), taking into account the non-covalent interactions of the rhodamine 6G and C4R1 molecules with subunits of ATP synthase. To search for the binding site, a small iterator was written using Python methods, taking a list of atoms on the surface of the subunit as the input, setting certain boundaries of local docking (cube 30*30*30 angstrom) centered in the coordinates of each atom from the list, and triggering the calculation of molecular docking. As a result, a set of conformations of rhodamine molecules associated with the investigated ATP synthase subunit was obtained. From all these sets, the conformation with the lowest energy value was selected.

## 3. Results

### 3.1. Uncoupling Activity of C4R1 in Mitochondria

The first goal was to confirm the uncoupling effect of C4R1, evaluated both by the activation of respiration in state 4 and by the effect on the maximal and ATP synthase-controlled respiration rate (states 3u and 3 correspondingly) of mitochondria isolated from rat liver.

**Figure 2:**
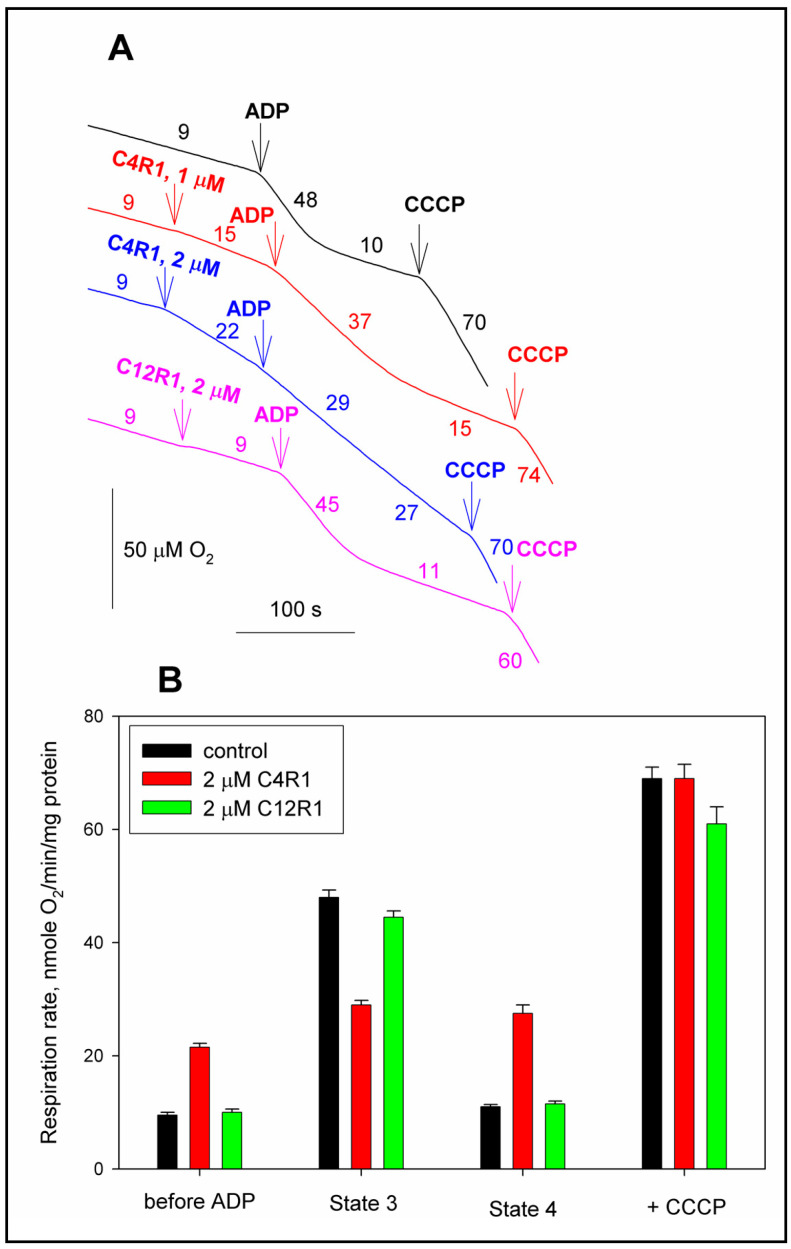
The effect of C4R1 and C12R1 on coupled (state 4), uncoupled (state 3u) and phosphorylated (state 3) respiration. (**A**) Traces of respiration of rat liver mitochondria (RLM). Substrate, succinate (5 mM) supplemented with 2 μM rotenone. Additions were indicated: 100 μM ADP, 0.2 μM CCCP. For other conditions, see the Materials and Methods. Numbers on curves correspond to mitochondrial respiration rates in nmoles O_2_/min/mg protein. (**B**) Statistics of the effect of C4R1 and C12R1 on the respiration of RLM. Results are expressed as mean ± SD (n = 3) with *p* < 0.001 (Student’s test) between black and red columns in groups “before ADP”, “State 3” and “State 4”.

Figure 2A shows that C4R1 used in concentration 1–2 µM activates state 4. On the other hand, the effect on initial respiration of its more hydrophobic analogue C12R1 used in the same concentrations was negligible while demonstrating a slightly diminished respiration rate at state 3. This data correlates with the previously shown one that the short-chain derivative of rhodamine 19 (C4R1) in the same concentrations as C12R1 causes a faster and more significant drop in the transmembrane potential of mitochondria [28].

### 3.2. The Effects of C4R1 and C12R1 in Bacillus subtilis Intact Cells

**Figure 3:**
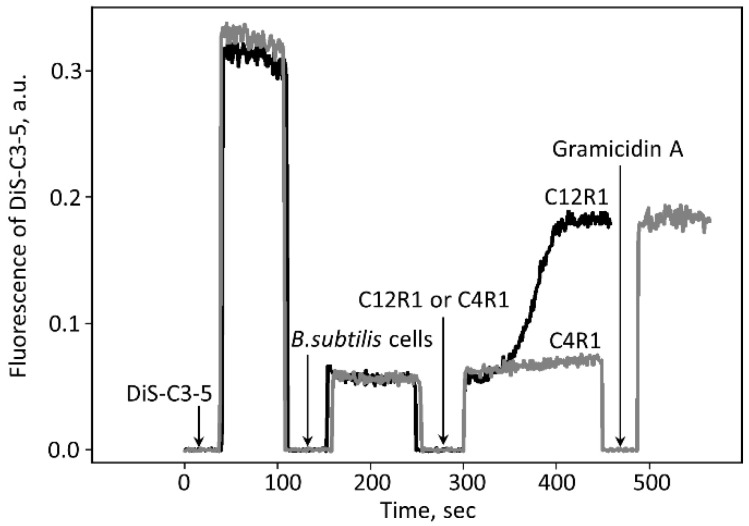
Changes in the electric potential across the membrane of *B. subtilis*. Changes in the membrane potential were monitored by measuring fluorescence of DiS-C3-(5) (10 μM) in PBS buffer. The short time intervals with the fluorescence falling to zero are caused by the opening of the chamber for making additions. C4R1 (0.6 µM) and C12R1 (0.6 µM) were added as shown. Gramicidin A (0.5 ng/mL) was added at the end of the experiment for perforation of the cell membrane to collapse the membrane potential.

The idea of the involvement of some mitochondrial membrane protein in the process of C4R1-mediated uncoupling was tested using another model, namely intact Gram-positive *Bacillus subtilis* cells. The addition of 0.6 µM C12R1 led to a decrease in the membrane potential of the bacterial cells, as judged by an increase in fluorescence of the potential-sensitive dye DiS-C3-(5) (Figure 3) known to be quenched by membrane potential [47,48,49].

On the other hand, C4R1 at the same concentration exhibited an almost insignificant effect. The action of C12R1 on the potential was delayed and characterized by slow kinetics depending on the concentration of C12R1 (Figure 3). Thus, in *B. subtilis* cells we observed the phenomenon fully opposite to that in mitochondria. This indicated that the mechanisms of uncoupling in mitochondria and *B. subtilis* cells are different.

### 3.3. Binding C4R1 and C12R1 with Mitochondria and Bacterial Cells

For a more detailed study of the mechanism of C4R1-induced uncoupling of mitochondria, we incubated isolated rat liver mitochondria with C4R1, after which the mitochondria were solubilized and subjected to electrophoresis in PAAG with subsequent detection of fluorescence in the gel.

Our attempts to use two-dimensional electrophoresis for this purpose failed and we were unable to detect fluorescence in any of the many electrophoretic spots detected by the Coomassie blue staining. On the other hand, we found that on the gel, all fluorescence was located in the frontal region near the maximum and minimum pH values (about 3 and 10, see Supplemental Figure S1). We must admit that in accordance with the technique of two-dimensional electrophoresis, in the first dimension there is a separation along the pH gradient, that is, from acidic values to more alkaline, and in the second dimension the separation goes according to the molecular size of proteins. We assumed that in the initial phase of the procedure, the release of the C4R1 binding to the protein occurs, resulting in the formation of free C4R1, which quickly goes into the frontal zone, and cleavage occurs at both low and high pH values. To confirm this, we used 1D electrophoresis and load a sample containing alkaline (NaOH with a final pH 10) to the gel well. Manipulation with the sample, such as heating, did not change the result which was negative: none of the bands in the resulting gel contained fluorescence confirming that an alkaline pH prevents C4R1 binding to any of the mitochondrial proteins.

**Figure 4:**
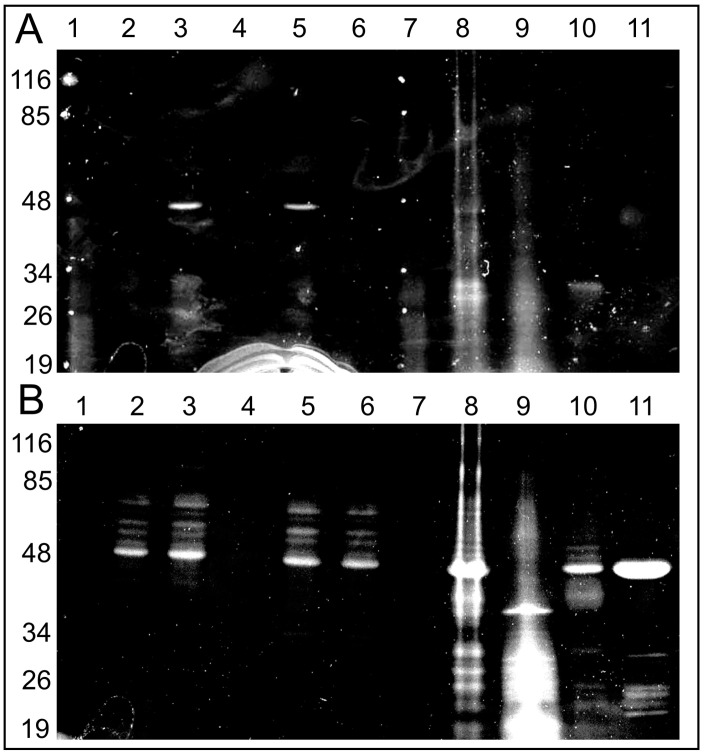
Fluorescent images of the gels after SDS electrophoresis of solubilized rat liver or heart mitochondria (RLM or RHM) exposed to C4R1 (5 µM) under different conditions (**A**). In (**B**), the same gel is shown but after its incubation with C4R1 (20 µM). Lanes: 1. MW markers; 2. RLM 40 µg protein; 3. RLM+C4R1; 4. RLM+C4R1+NaOH; 5. RLM+C4R1+FCCP; 6. RLM+FCCP; 7. MW markers; 8. frozen RHM 40 µg protein. No dye; 9. Fraction 1800 g of RHM. No dye; 10. Fraction 8000 g of RHM. No dye; 11. Isolated ATP synthase. No dye.

However, if a neutral pH was maintained in the mitochondrial sample (pH 7.2), we were able to observe few fluorescing bands with obvious major band around 48–50 kDa after 1D electrophoresis. The experiment was executed in two ways: the first run was with mitochondria incubated under different modes: (1) without C4R1; (2) with the dye; (3) with the dye in the presence of an uncoupler to exclude the effect of the membrane potential; and (4) with the dye in the presence of alkali (Figure 4). Two mitochondrial preps were used: coupled rat liver mitochondria (RLM) and rat heart mitochondria (RHM). In addition, in one electrophoretic well, we placed a prep of pure ATP synthase isolated from a rat heart. Then, the same gel was soaked with free C4R1 (20 µM) for 30 min and after this, we were able to detect the fluorescing 48–50 kDa component in all samples except that treated with alkali (Figure 4).

Importantly, pure isolated ATP synthase complex binds C4R1 with an obviously major component having a molecular mass around 48–50 kDa.

Altogether, this data pointed to the fact that some components of the ATP synthase complex (Complex V) with a molecular mass in the range 48–50 kDa (e.g., α and/or β subunits of F1 part of Complex V) bind C4R1. High pH sensitivity of the binding indirectly pointed to its electrostatic nature.

**Figure 5:**
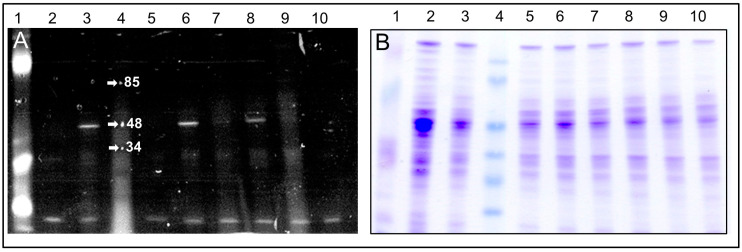
Images of the gels after SDS electrophoresis of solubilized mitochondria exposed to C4R1 or C12R1 under different conditions. (**A**), fluorescence; (**B**), the same gel stained with Coomassie blue. Lanes: 1. MW fluorescent markers; 2. RLM 40 µg; 3. RLM+C4R1 (5 µM); 4. MW markers; 5. RLM; 6. RLM+C4R1 (5 µM); 7. RLM+C12R1 (5 µM); 8. RLM+ C4R1(5 µM) + T3 (20 µM); 9. RLM+C4R1 (5 µM)+T3 (20 µM); 9. RLM+T3 (20 µM); 10. RLM. Note the low MW autofluorescence band on the bottom of the gel supporting equal protein loading in wells.

We found that C4R1 binding to the 48–50 kDa component was insensitive to the presence of thyroid hormone T3 (triiodothyronine) which also belongs to mild uncouplers indicating a non-competitive character of action of these two compounds (Figure 5).

**Figure 6:**
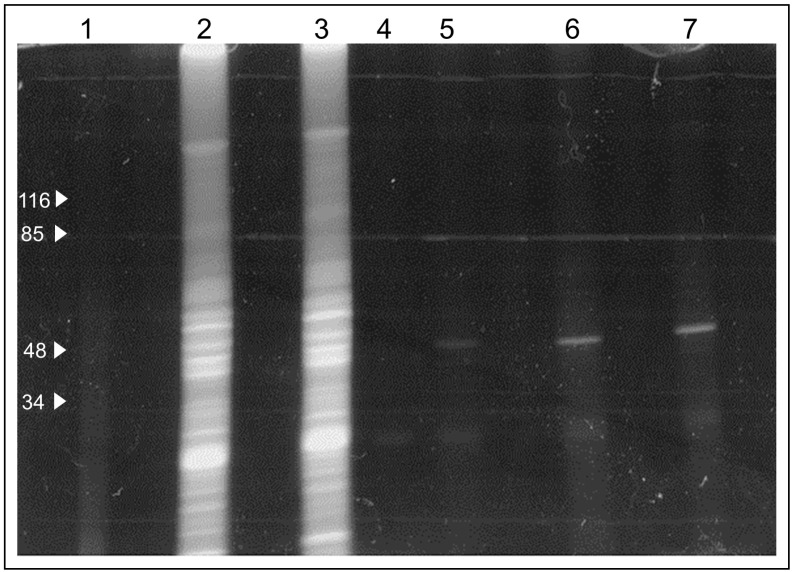
Fluorescent Images of the gel after SDS electrophoresis of solubilized rat liver mitochondria (RLM) exposed to SkQR1, C4R1 and C12R1 conditions. Lanes: 1. MW markers; 2. SkQR1 (5 µM); 3. SkQR1 (5 µM); 4. RLM; 5. RLM+C12R1 (5 µM); 6. RLM+C4R1 (5 µM)+2,4 dinitophenol (100 µM); 7. RLM+C4R1 (5 µM).

We also compared the mitochondrial binding efficiency of C4R1, C12R1 and another rhodamine derivative carrying antioxidative moiety namely SkQR1 (for structure, see [29]). In contrast to the strong binding of C4R1 to the 48–50 kDa band, SkQR1 had many mitochondrial proteins to bind with and there was a faint binding of C12R1 to the 48–50 kDa band (Figure 6).

**Figure 7:**
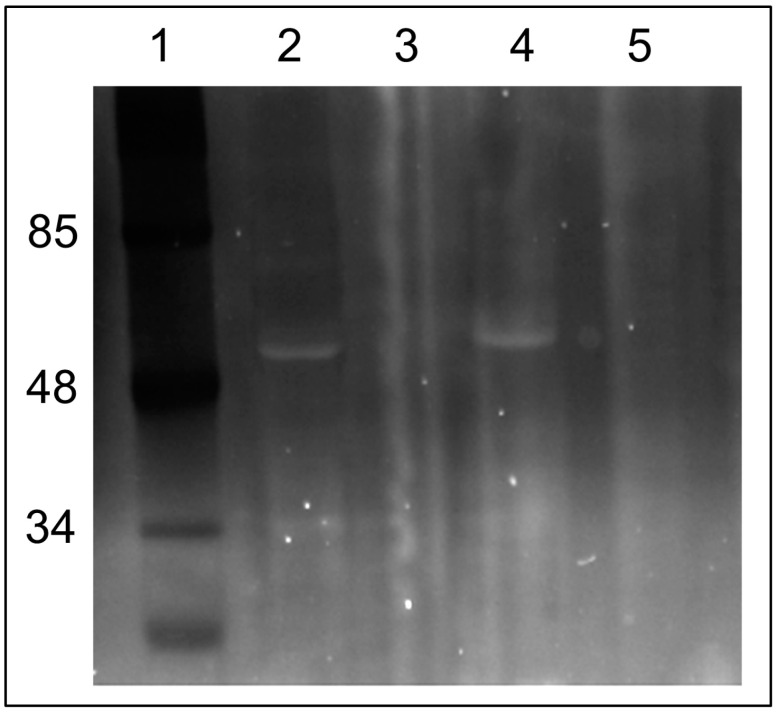
Fluorescent image of the gel after SDS electrophoresis of solubilized rat liver mitochondria (RLM) or extracts of *B. subtilis* exposed to C4R1 (5 µM). Lanes: 1. MW markers; 2. RLM+C4R1; 3. Blank well; 4. RLM+C4R1; 5. *B. subtilis*+C4R1. Note the fluorescing band in RLM extracts at around 50 kDa.

Next, we tested whether the extracts from *B. subtilis* bind C4R1 considering that uncoupling activity of C4R1 in these cells was low (see, Figure 3) in contrast to C12R1 demonstrating high efficiency in lowering the membrane potential of these bacterial cells. We found that binding of C4R1 to *B. subtilis* was negligible (Figure 7).

### 3.4. Identification of the Mitochondrial Component That Binds C4R1

The next goal was to clearly identify the nature of the 48–50 kDa component. To do this, we excised from the gels fluorescing bands of different regions and examined their content by mass spectrometry. One sample contained all fluorescing bands from the region covering molecular masses of 23–50 kDa. In this mixture, a total of 97 peptides were identified with a major contribution of ATP synthase subunit beta and ATP synthase subunit alpha with a mascot score of 517. The highest single protein hit was ATP synthase subunit beta with the number of mass values matched being 51 which covered 87% of its sequence. For ATP synthase subunit alpha, the score was 160, the number of mass values matched was 30 which covered 49% of its sequence.

We must admit that in the gel, we could not resolve the binding of a probe to α or β subunits since visually we detected a single fluorescing band in this region. However, for accurate detection from the fluorescent gel, we cut two strips in accordance with the Coomassie staining, corresponding to 48 kDa (presumably belonging to the β-subunit) and 50 kDa (presumably, belonging to α subunit of ATP synthase) bands. Indeed, these two bands contained either α-subunit (mascot score 252, number of peptides:34, sequence coverage 49%) or β-subunit (mascot score 417, number of peptides:56, sequence coverage 87%).

Ultimately, this indicates that ATP synthase subunits α and β were indeed the proteins abundant in selected gel bands (mascot analysis is presented in the archive of the Supplement).

### 3.5. Modelling of C4R1 Binding to Mitochondrial Component

**Figure 8:**
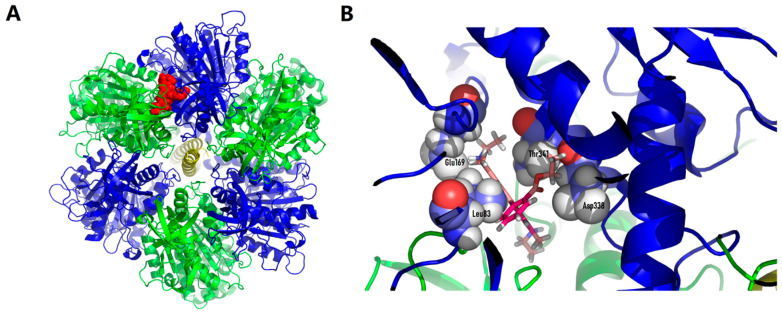
Rhodamine 6G docked structures on human ATP synthase. (**A**), whole ATPase complex overview (green, α-subunits of ATP synthase; blue, β-subunits of ATP synthase; red, rhodamine 6G). (**B**), binding pocket close up look with amino acid residues (L83, E169, D338, T341) involved in binding to rhodamine molecule. While searching for the binding site during docking from several locations, the equally optimal location for the ligand was found. The estimated binding energy was estimated by the Autodock vina program at 8.3 kcal/mol.

First, we created a model of the specific binding of Rhodamine 6G (which is structurally similar to C4R1 and can be named C2R1) to the β-subunit of the ATP synthase. The simulation took into account that rhodamine molecule binds to mitochondria, and not to *B. subtilis* (Figure 8). We assumed that the different binding mode was caused by a difference in the structure of the β-subunit of the ATP synthase.

**Figure 9:**
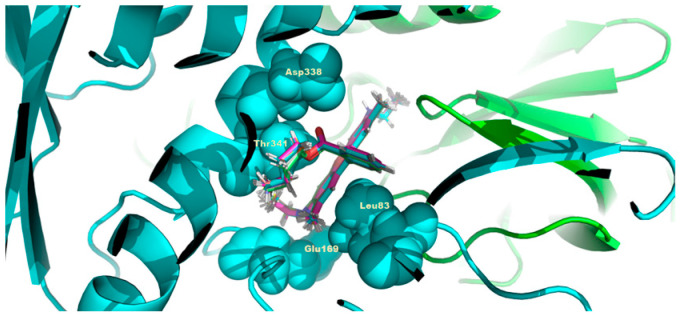
C4R1 docked structures on human ATP synthase. The conditions are the same as in Figure 8 except that the α-subunit of ATP synthase is shown in turquoise and β-subunit of ATP is in light green. C4R1 molecule is locked in the center of the figure entrapped in the same pocket as for C2R1 organized by the same amino acid residues (L83, E169, D338, T341).

The next step was to represent the binding locus on the ATP synthase structure for the molecule, which was investigated using the approaches outlined above for C2R1 (Rhodamine 6G), that is, the C4R1 molecule (Figure 9). As expected, the binding was at the same locus of ATP synthase with the participation of the same amino acid residues that participated in the binding of C2R1. Thus, this locus on the α-subunit is to some extent universal for binding analogues of rhodamine 6G, including C4R1.

Figure S2 shows the alignment of the structures of the β-subunit of the mitochondrial and bacterial ATP synthase. The fact that binding was very sensitive to pH provides convincing evidence of the electrostatic interaction of positively charged rhodamine molecule with a negatively charged region on the β-subunit. Ultimately, we outlined a few negatively charged specific sites that differs in mitochondria and *B. subtilis* which in general meets the criteria of the model.

## 4. Discussion

In this work, we revealed the association of the uncoupling action of the penetrating C4R1 cation with the predominant binding of this agent to the component of the ATP synthase complex, namely the β-subunit of the F1 component. Although we have not established a causal relationship between binding and uncoupling; nevertheless, given that the binding of C4R1 to other components than mitochondrial ATP synthase was minor, we assumed that it was ATP synthase that could be responsible for mild uncoupling, and our assumption is supported by basic knowledge about the mechanisms of uncoupling.

The study of the uncoupling mechanism is primarily determined by practical interest, because in various pathological situations, the beneficial effect of a slight decrease in the mitochondrial membrane potential has been proven, contributing to the mild course of pathology and a better outcome. Such a positive effect, in particular, has been shown in an injured brain and kidney [26,27,28,29,31,38,40].

By its principle, uncoupling of oxidative phosphorylation results in a lower efficiency of ATP-synthetic mitochondrial machinery. The efficiency of mitochondrial ATP synthesis is determined by both the intrinsic properties of ATP synthase depending on its working architecture [50] and from the extrinsic properties of the mitochondrial inner membrane affording an active or passive ion leak compromising the membrane potential [51].

Generally speaking, the activation of respiration is not always associated with the drop in the membrane potential, at least in living cells. An example is taken from the diazoxide-induced activation of respiration in cardiac myocytes, which was not associated with a drop in membrane potential, i.e., not through effective uncoupling. This higher respiration was interpreted as an accelerated respiratory flux, and because of this, it was associated with higher ROS production [52,53]. Although, strictly speaking, diazoxide, which is the opener of the ATP-dependent K-channel, cannot easily be considered as a mild uncoupler; however, in a practical sense it can. Theoretically, any mild uncoupler could do the same thing—activate respiration without changing the membrane potential while increasing the total production of ROS. This situation is unlikely to be accomplished in isolated mitochondria, but in a living cell with a high content of ATP, which can be used to maintain the membrane potential of mitochondria, it does not look improbable.

If such a scenario is allowed, then a moderate uncoupling can not only reduce but also increase ROS production. The latter can be achieved by activating proton pumping and increasing oxygen flux in parallel with higher ROS production, given that a constant part of the oxygen consumption by mitochondria is utilized for ROS production. This could compensate for the drop in membrane potential caused by an ion leak, providing a stable membrane potential. The possible conflict of the data obtained with isolated mitochondria and living cells makes it necessary to be very careful when interpreting the data obtained in isolated mitochondria [53]. Considering all these arguments, the beneficial effect of mild uncoupling can be associated not (exclusively) with changes in ROS levels, but also with other components resulting from the activation of respiration, such as an increased production of CO_2_ and H_2_O and higher utilization of substrates (for example, fat).

CO_2_ is known as an essential physiological regulator of cellular metabolism [54,55,56,57,58,59,60,61]. Recent data indicates that bicarbonate may also be involved in the mechanism of mitochondrial membrane potential stability [61], which indirectly demonstrates an obvious relationship between CO_2_ levels and the uncoupling mechanism. Water is another important product of the uncoupling process, and even its slight increase in the mitochondrial matrix can activate mitochondrial metabolism through the so-called “regulatory swelling of mitochondria” [52,53], which is an integral part of the cell protective mechanism.

In addition, we must admit that the mild ROS burst (at least temporarily) can also be useful due to its participation in the protective mechanism of ischemic preconditioning [53].

Whatever the source of the useful mechanism of mild uncoupling, our data is in support of that the ATP synthase complex can be considered as belonging to more extensively determined uncoupling proteins. Our data on the binding of the fluorescent agent C4R1, which confirmed its properties of the mild uncoupler in experiments using isolated mitochondria directly indicated the participation of ATPase in the process of uncoupling. We repeat that this study is so far limited to the statement that the binding of C4R1 to ATP synthase is associated with subsequent uncoupling, without insisting on the presence of a cause–effect relationship. It should be noted that data of this study indicates that binding occurs exclusively with alpha and beta structures of the ATPase, and other components of the ATP synthase show only very slight binding to C4R1; however, the participation of components such as gamma stalk and delta subunits can certainly be highly probable. Possibly, other, if not all, components of ATP synthase complex might be involved in the resulting uncoupling because, without a doubt, a proton leak preferably goes through the structure in Fo [3] which contains numerous components, and additional work is needed to uncover the exact mechanism of C4R1 (and may be other drugs)-induced uncoupling. Additionally, we must admit that there is a conflict of ideologies on the functioning of ATP synthase: while one [62,63] insisted that there is a slip in the rotation of the c-subunit disk in the membrane, alternative opinions excluded leak and slip during the normal operation of ATP synthase [50,64,65]. In this work, ATP synthase was not under normal operation mode, which is caused by the binding of a mild uncoupler. We assume that switching from the normal fully coupled mode of operation of ATP synthase to the leaking mode of operation occurring after binding with the uncoupling agent is due to a change in the chemical interaction of Fo and F_1_. Thus, many components of Fo may take part in the binding-related uncoupling mechanism, but a proper assessment of such an interaction will require additional extensive work.

We should note that in our argumentation, we do not take into account the lability of the structure of the ATP synthase complex, in particular its state of dimerization-monomerization, which, according to existing concepts, takes part in the organization of the mitochondrial pore, that is mitochondrial permeability transition (reviewed in [66]). First, uncoupling and permeability transition are different phenomena and secondly, the change in ultrastructural conformations under these conditions goes in different directions, leading to swelling under induction of permeability transition and condensation of the matrix under uncoupling, which differently affects the conformation of the mitochondrial cristae of the inner membrane of mitochondria, possibly participating in the dimerization-monomerization process [66].

We have also excluded from consideration the regulatory role of guanine nucleotides, which are extremely important in the regulation of uncoupling proteins of the UCP family [23], but do not play a role in uncoupling going with the participation of other proteins that are not structurally homologous to the UCPs [12,13,14,15].

Finally, we must note that in the seventies of the last century, based on studies with an affinity-labeling uncoupler, the evidence was obtained that mitochondria contain a specific uncoupler binding site (protein of 30 kDa), which is capable of ATP-Pi exchange, suggesting that this site is located in complex V [67].

## 5. Conclusions. Uncoupling Proteins: One More?

In this study, we confirmed that the process of uncoupling of oxidative phosphorylation in mitochondria can be mediated by mitochondrial proteins residing in the inner membrane of mitochondria. Recently, the old dogma that it is enough to have only a bilayer phospholipid membrane and small molecules that can be protonated on the one side of the membrane and release protons on the other side has become the subject of revision. According to this dogma, this mechanism enables the removal of the proton gradient and it generally leads to the drop in the transmembrane potential of hydrogen ions generated by proton pumps of the inner membranes of mitochondria. Existing data indicates that in addition to this mechanism, there is another one that ensures the transfer of protons through the inner mitochondrial membrane with the help of proteins inhabiting this membrane. Prior to this work, such a collection of uncoupling proteins included true uncoupling proteins from the UCP family, an adenine nucleotide translocator and a dicarboxylate transporter. We propose to supplement this collection with an ATP synthase complex, which can provide a discharge of the membrane potential associated with the binding of a cationic dye from the rhodamine family.

## Data Availability

The data that support the findings of this study are available from the corresponding author upon reasonable request.

## Supplementary Materials

The following supporting information can be downloaded at: https://www.mdpi.com/article/10.3390/antiox12030646/s1, Figure S1: 2D electrophoresis of extract of rat liver mitochondria incubated with C4R1 (A, B) and 1D electrophoresis (C, D) of extract kept at pH10. Arrows show the areas containing fluorescence. In C, left lane, MW markers, and other two lanes contained mitochondrial samples (80 and 40 µg protein/per well correspondingly). Coomassie staining.; Figure S2: Alignment of β subunits of ATP synthase of Homo sapience, Escherichia coli, Bacillus subtills and Rattus norvegicus, predicted binding residues are marked with red boxes.

## Abbreviations

C4R1, rhodamine 19 butyl ester; C12R1, rhodamine 19 dodecyl ester; FCCP, carbonyl cyanide p-trifluoromethoxyphenylhydrazone; CCCP, carbonyl cyanide m-chlorophenylhydrazone; UCP, uncoupling protein; ROS, reactive oxygen species; RLM, rat liver mitochondria; RHM, rat heart mitochondria.
