# Supplementary material for: Mitochondrial ATP Synthase and Mild Uncoupling by Butyl Ester of Rhodamine 19, C4R1
Source: Antioxidants (Basel). 2023 Mar 4;12(3):646. doi: 10.3390/antiox12030646 (PMC10044837; doi:10.3390/antiox12030646)
Supplement: Supplementary file 1 [file antioxidants-12-00646-s001.zip › Suplemental figures.pdf]

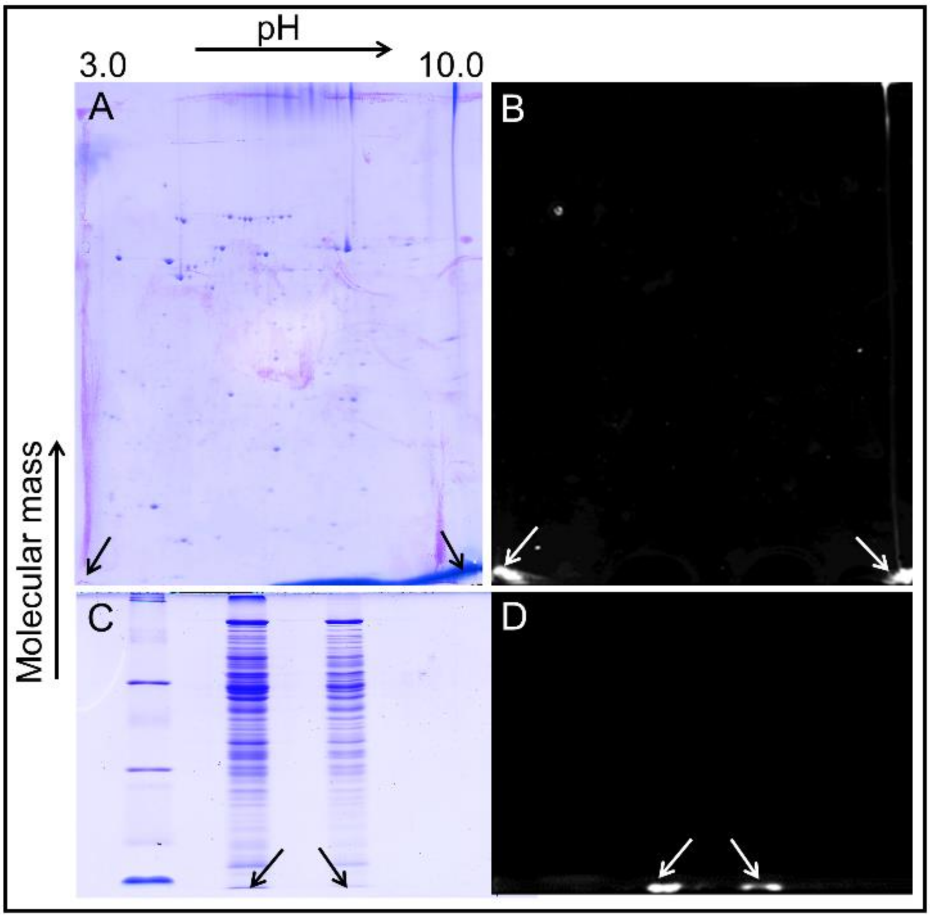

Figure S1. 2D electrophoresis of extract of rat liver mitochondria incubated with C4R1 (A, B) and 1D electrophoresis (C, D) of extract kept at pH10. Arrows show the areas containing fluorescence. In C, left lane, MW markers, and other two lanes contained mitochondrial samples (80 and 40 µg protein/per well correspondingly). Coomassie staining.

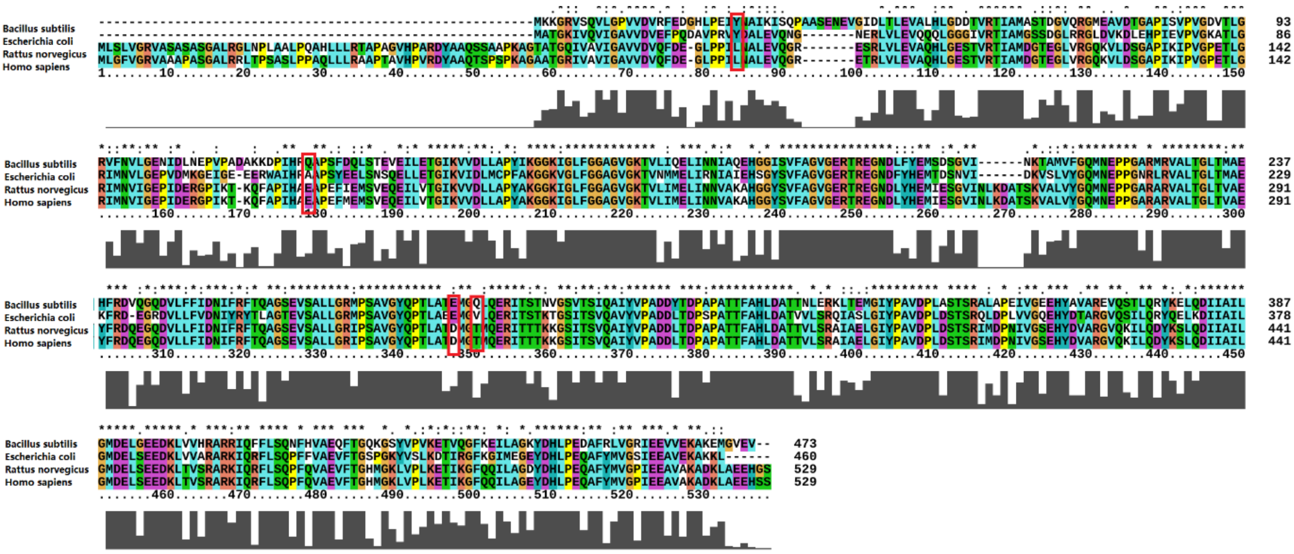

Figure S2. Alignment of β subunits of ATP synthase of *Homo sapience, Escherichia coli, Bacillus subtills* and *Rattus norvegicus*, predicted binding residues are marked with red boxes.
